# Superior Capacitive Energy Storage of BaTiO_3_‐Based Polymorphic Relaxor Ferroelectrics Engineered by Mesoscopically Chemical Homogeneity

**DOI:** 10.1002/advs.202502916

**Published:** 2025-05-24

**Authors:** Aiwen Xie, Ziyi Yu, Junwei Lei, Yi Zhang, Ao Tian, Xuewen Jiang, Xinchun Xie, Yuewei Yin, Zhenqian Fu, Xiaoguang Li, Ruzhong Zuo

**Affiliations:** ^1^ Center for Advanced Ceramics School of Materials Science and Engineering Anhui Polytechnic University Wuhu 241000 P. R. China; ^2^ Anhui Key Laboratory of Low Temperature Co‐fired Materials School of Chemistry and Materials Engineering Huainan Normal University Huainan 232038 P. R. China; ^3^ State Key Laboratory of High Performance Ceramics and Superfine Microstructures Shanghai Institute of Ceramics Chinese Academy of Sciences Shanghai 200050 P. R. China; ^4^ Hefei National Laboratory for Physical Sciences at the Microscale Department of Physics and CAS Key Laboratory of Strongly‐Coupled Quantum Matter Physics University of Science and Technology of China Hefei 230026 P. R. China

**Keywords:** energy storage capacitors, ex‐/in situ multiscale structure evolution, mesoscopically chemical homogeneity, polymorphic polar nanodomains, relaxor ferroelectrics

## Abstract

Relaxor ferroelectrics exhibit giant potentials in capacitive energy storage, however, the scales of polar nanoregions determine the critical field values where the polarization saturation occurs. In this work, a mesoscopic structure engineered ergodic relaxor state is realized by adjusting submicron‐grain scaled chemical homogenity, exhibiting polymorphic polar nanoregions of various scales in different grains. This produces a relatively continuous polarization switching with increasing the applied electric field from diverse grains, thus resulting in a linear‐like polarization response feature. As a result, both a giant energy density (*W_rec_*) ≈15.4 J cm^−3^ and a field‐insensitive ultrahigh efficiency (*η*) ≈93.2% are simultaneously achieved at 78 kV mm^−1^ in (Ba, Ca)(Ti, Zr)O_3_‐(Bi_0.5_Na_0.5_)SnO_3_ lead‐free ceramics. Moreover, both the mesoscopic structure heterogeneity and complex high internal stresses in ultrafine grains decrease the temperature sensitivity of the nanodomain structural features. Together with the suppressed high‐temperature defect motion from high ceramic density and submicron grain size, a record‐high temperature stability with *W_rec_* = 10.4±5% J cm^−3^ and *η* = 96±3% is obtained at 65 kV mm^−1^ and 0–250 °C, demonstrating great application potential of the studied ceramic in high‐temperature energy storage capacitors. The proposed strategy in this work greatly expands the design mentality for next‐generation high‐performance energy‐storage dielectrics.

## Graphical abstract

BaTiO_3_‐based polymorphic relaxor ferroelectrics are engineered by mesoscopically chemical homogeneity, constructing diversified nanodomain architectures with similar multiple local symmetries but different nanodomain scales in diverse grains. Ex‐/In situ multiscale structure analysis indicates that a relatively continuous polarization switching with increasing the applied electric field from the diverse grains is responsible for the superior energy‐storage properties.

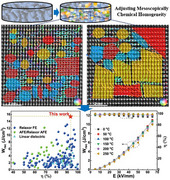

## 1 Introduction

High‐performance dielectric energy‐storage capacitors are pressingly required in diverse applications, including the storage of renewable energy, electronic devices, and pulsed power supplies.^[^ ^1^, ^2^, ^3^, ^4^, ^5^ ^]^ Compared with electrochemical energy‐storage systems, dielectric energy‐storage capacitors feature ultrahigh power density (*P_D_*), ultrafast charge/discharge rate, and good reliability, demonstrating strong competitiveness for efficient and reliable energy storage. However, their stubborn inadequacy of relatively low energy‐storage capability becomes an issue during gradually ascensive usage requirements.^[^ ^6^, ^7^, ^8^, ^9^, ^10^, ^11^, ^12^ ^]^ Moreover, the consideration of environmental protection requires the use of environmental‐friendly dielectric materials.^[^ ^7^, ^8^, ^9^, ^10^, ^11^, ^12^ ^]^ Consequently, considerable research work has been devoted into developing lead‐free dielectrics with simultaneously high recoverable energy‐storage density (*W_rec_*) and efficiency (*η*).^[^ ^13^, ^14^, ^15^, ^16^, ^17^ ^]^

The energy‐storage ability of a dielectric is mainly determined by its polarization‐field response traits, and the concurrent achievement of the delayed polarization saturation, low polarization hysteresis, and large *P_max_* is widely recognized as a goal pursued for simultaneously high *W_rec_* and *η* values.^[^ ^6^, ^7^, ^8^, ^9^, ^10^ ^]^ Relaxor ferroelectric (FE) materials thus become preferred options for high‐performance dielectric capacitors owing to the relatively low polarization hysteresis from highly‐dynamic polar nanoregions (PNRs).^[^ ^6^, ^7^, ^8^, ^9^, ^10^ ^]^ Generally, the transformation from long‐range ordered micrometer‐sized domains in normal FEs into short‐range ordered nanodomains in relaxor FEs is derived from enhanced local composition heterogeneity. As a result, reducing domain size into nanoscale has become a commonly‐used way to effectively improve energy‐storage properties of dielectrics.^[^ ^5^, ^6^, ^7^, ^8^, ^9^, ^10^ ^]^ However, even in a superparaelectric relaxor state displaying nanodomains with a scale of only several unit cells, the inevitable growth process of nanodomains under a sufficiently high electric field always creates a mutual constraint between *P_max_* and polarization hysteresis.^[^ ^17^, ^18^ ^]^ Designing polymorphic nanodomains with the coexistence of multiple local FE symmetries has recently been reported to effectively improve the contradiction by smoothing the domain‐switching way, making great progress in energy storage properties of relaxor FE films and ceramics.^[^ ^12^, ^19^, ^20^, ^21^ ^]^ Nevertheless, a compromise between *W_rec_* and *η* at a moderate electric field is still a common choice in most current works because that the increase of *W_rec_* always accompanies a declined *η* under higher electric fields owing to the unaltered domain growth behavior. This has become a bottleneck for further breakthroughs in the energy‐storage performance of dielectric capacitors.

**Figure 1:**
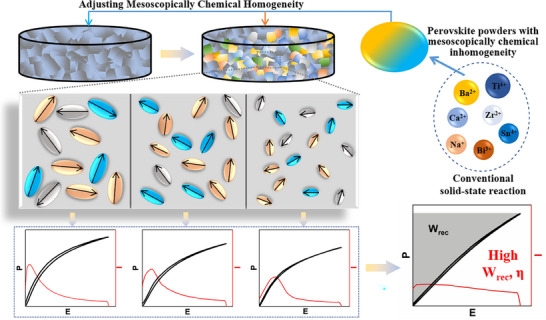
A diagrammatic sketch of achieving superior capacitive energy‐storage performances in BT‐based polymorphic relaxor FE ceramics engineered by mesoscopically chemical homogeneity. The different colored areas with arrows represent various PNRs with different local FE symmetries.

In the present study, we proposed a novel strategy to further enhance the energy storage performance of the polymorphic relaxor FEs by adjusting msoscopically chemical homogenity, as shown in **Figure** **1**. Coexistence of multiple local FE symmetries can thus be engineered in the chemically heterogeneous grains as PNRs with different scales. The PNRs with various scales possess different driving electric fields for the transformation toward long‐range ordered FE domains,^[^ ^22^, ^23^, ^24^ ^]^ thus producing a realtively continuous polarization switching with increasing the applied electric field from the diverse grains. This would lead to a macroscopically highly‐diffused field‐induced relaxor to FE phase transition, which can effectively overcome the contradiction between *P_max_* and polarization hysteresis. As a result, a linear‐like polarization‐field response is expected to be realized, being in favor of simultaneously achieving high *W_rec_* and *η* values. The above strategy can be realized in BaTiO_3_ (BT)‐based compositions at rhombohedral (R)‐orthorhombic (O)‐tetragonal (T)‐cubic (C) region.^[^ ^25^, ^26^, ^27^, ^28^ ^]^ BT‐based ceramics were selected for its tunable polycrystalline phase boundary between multiple FE phases and absence of high‐temperature volatile ions as compared with (Na, K)NbO_3_. By introducing diverse ions with significant differences in diffusion rates during sintering, the calcined powders can possess a single perovskite structure and chemical inhomogeneity simultaneously. The chemical inhomogeneity can thus be maintained in spark plasma sintered (SPS) ceramics due to the fast sintering process.^[^ ^23^, ^29^ ^]^ Notably, owing to the significant refinement of grain size, SPS ceramics would also exhibit high dielecric breakdown strength (*E_B_*),^[^ ^13^ ^]^ being beneficial for exploiting the advantages of this strategy for energy storage under ultrahigh external fields. Consequently, our designed BT‐based ceramics simultaneously realize a giant *W_rec_* of msoscopically 15.4 J cm^−3^ and an ultrahigh *η* of msoscopically 93.2% under a high electric field of msoscopically 78 kV mm^−1^, making breakthroughs of comprehensive energy‐storage properties in lead‐free ceramics.

## 2 Results and Discussion

**Figure 2:**
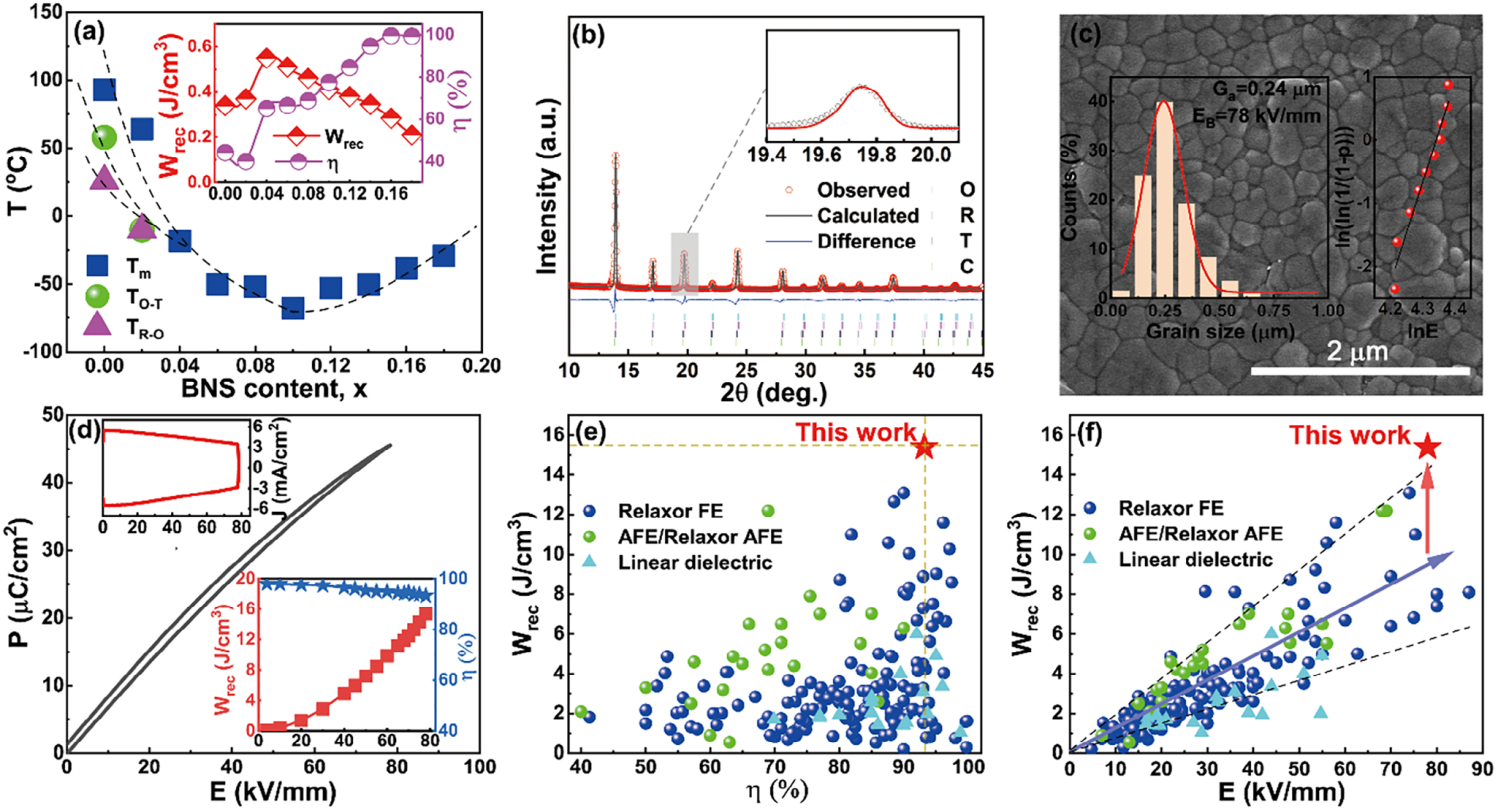
a) The composition‐structure‐temperature phase diagram for the (1‐*x*)BCZT‐*x*BNS conventionally sintered ceramics. b) Rietveld SXRD refinement results for the *x* = 0.16 SPS ceramic powders. c) SEM images of the *x* = 0.16 SPS ceramics. The insets of (c) show the grain size distribution and Weibull distribution of the *E_B_* values. d) Room‐temperature *P‐E* loops measured under the maximum testable electric fields and 10 Hz and the insets of (d) show the corresponding polarization current density‐electric field (*J‐E*) curve and field‐dependent *W_rec_* and *η* values of the *x* = 0.16 SPS ceramics. e‐f) Comparisons of room‐temperature energy‐storage properties between the *x* = 0.16 SPS samples and other recently reported lead‐free bulk ceramics.^[^ ^4^, ^5^, ^6^, ^7^, ^8^, ^9^, ^10^, ^11^, ^12^, ^13^, ^16^, ^18^, ^19^, ^20^, ^21^, ^22^, ^30^, ^31^, ^32^, ^33^, ^34^, ^35^ ^]^

A polymorphic phase boundary between R, O, and T FE phases in BT‐based ceramics can be easily constructed by shifting the polycrystalline phase transition (PPT) temperatures close to room temperature through composition adjustment.^[^ ^25^, ^26^, ^27^, ^28^ ^]^ As shown in **Figure** **2**a, the curie temperature *T_c_*, R‐O (*T_R‐O_*), and O─T (*T_O‐T_*) PPT temperatures of the (1‐*x*)Ba_0.94_Ca_0.06_Zr_0.9_Ti_0.1_O_3_‐*x*(Bi_0.5_Na_0.5_)SnO_3_ ((1‐*x*)BCZT‐*x*BNS) solid solutions prepared by a conventional sintering (CS) method can be found to approach each other with increasing BNS content, leading to the construction of a quadruple phase zone where C, R, O, and T phases converge together at *x* ≥ 0.04, which can be evidenced by the temperature‐dependent dielectric properties and Rietveld refinement results on SXRD data (Figure S1a–c, Supporting Information). This process also accompanies an enhanced dielectric relaxor behavior, as confirmed by the increased degree of diffuseness (*γ*) and the relaxation factor *ΔT_relax_* (= *T_m,1_* *_MHz_* – *T_m,1_* *_kHz_*), as shown in Figure S1d, Supporting Information. The *x* = 0.16 composition was chose as a case study to realize our design owing to its excellent overall energy‐storage properties, as shown in the inset of Figure 2a. A solid‐state reaction synthesis method together with SPS technique was used to realize the high density, fined grains as well as mesoscopic chemical inhomogeneity of *x* = 0.16 ceramic samples owing to an extremely‐short sintering time.^[^ ^23^, ^24^, ^25^, ^29^ ^]^ The *x* = 0.16 SPS ceramics exhibit a pure perovskite structure with R‐O‐T‐C phases coexistence according to the Rietveld refinement results on SXRD data (Figure 2b), and an extremely samll average grain size (G_a_) of ≈0.24 µm and a high sample density of >99% lead to a high *E_B_*,^[^ ^13^, ^22^ ^]^ as shown in Figure 2c. Moreover, a broad dielectric anomaly peak with a large *ΔT_relax_* of ≈69 °C and a high *γ* of 1.95 indicates a strong dielectric relaxor behavior, as shown in Figure S1f, Supporting Information. The polarization versus electric field (*P‐E*) loop and corresponding polarization current density‐electric field (*J–E*) curve measured under the maximum testable field as well as the field‐dependent *W_rec_* and *η* values of the *x* = 0.16 SPS ceramics are displayed in Figure 2d. Only a polarization current platform instead of a detectable current peak can be observed from the *J‐E* curve, indicating a continuously increased polarization magnitude with electric fields. As a result, a low polarization hysteresis and a delayed polarization saturation can be obtained, leading to a linear‐like *P‐E* loop. It is technically significant that a giant *W_rec_* of ≈15.4 J cm^−3^ and a superior *η* of ≈93.2% are simultaneously achieved in the *x* = 0.16 SPS ceramics under 78 kV mm^−1^, which are superior to those of most currently reported lead‐free energy‐storage ceramics,^[^ ^4^, ^5^, ^6^, ^7^, ^8^, ^9^, ^10^, ^11^, ^12^, ^13^, ^16^, ^18^, ^19^, ^20^, ^21^, ^22^, ^30^, ^31^, ^32^, ^33^, ^34^, ^35^ ^]^ as shown in Figure 2e. Notably, it can be found from the relationship between *W_rec_* and its corresponding test electric fields (Figure  2f) that the *W_re_*_c_ value of most lead‐free relaxor FE ceramics has an approximately linear relationship with the test electric field, which can be broken in the BT‐based relaxor FEs in our current work. This indicates the significant contribution of factors other than the improved *E_B_* to the enhancement of energy storage performance. Moreover, to reveal the actual energy discharge behavior, underdamped and overdamped pulsed‐discharge electric current‐time (*I–t*) curves were also measured under different electric fields, as shown in Figure S2, supporting information. A short discharge time *t_0.9_* (the time required for releasing 90% of the stored energy) of <50 ns is consisted with the fast polarization response. A large power density *P_D_* of ≈326 MW cm^−3^ and a high discharge energy density *W_D_* of ≈5.8 J cm^−3^ obtained at 50 kV mm^−1^ illustrate outstanding charge‐discharge performances compared with most lead‐free dielectric ceramics, as shown in Figure S2e (Supporting Information).

**Figure 3:**
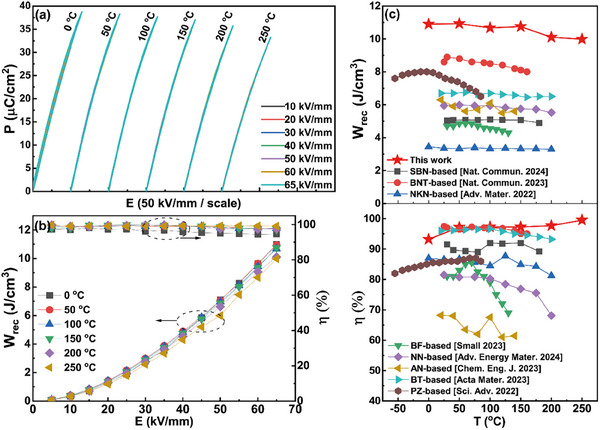
a) Electric field‐/temperature‐dependent *P‐E* loops measured at 10 Hz and b) evolution of *W_rec_* and *η* values with electric field and temperature for the SPS ceramics. c) The comparison of thermal stability of *W_rec_* and *η* values between the SPS ceramic and various recently‐reported representative energy‐storage ceramics.^[^ ^11^, ^12^, ^37^, ^38^, ^39^, ^40^, ^41^ ^]^

The thermal stability of the energy storage performance is also of vital importance since its deterioration at high temperatures severely restricts the operating temperature range of current dielectric capacitors.^[^ ^11^, ^12^ ^]^ Therefore, electric field‐dependent *P‐E* loops measured at different temperatures of the *x* = 0.16 SPS ceramic are shown in **Figure** **3**a. A steady linear‐like polarization response with extremely‐low hysteresis against both electric field and temperature can be observed, which is responsible for the electric/temperature field‐insensitive high *η* values, as shown in Figure 3b. It is worth noting that the *η* value increases from ≈94% at 0 °C to ≈99% at 250 °C. This should be related to the easier polarization reorientation under electric fields from enhanced nanodomain dynamic on heating, accounting for the reduced polarization hysteresis. In this way, the increased *η* compensates for the decrease in *W_rec_* caused by the decreased *P_max_* at high temperatures, leading to a good thermal stability of *W_rec_* values, as shown in Figure 3b. As a result, both a large *W_rec_* value of 10.4 ± 5% J cm^−3^ and a superb *η* value of 96% ± 3% can be obtained at 65 kV mm^−1^ within an ultra‐broad temperature range of 0–250 °C, indicating obvious advantages in high‐temperature energy‐storage capacitor applications as compared with various recently‐reported energy‐storage ceramics.^[^ ^11^, ^12^, ^37^, ^38^, ^39^, ^40^, ^41^ ^]^

**Figure 4:**
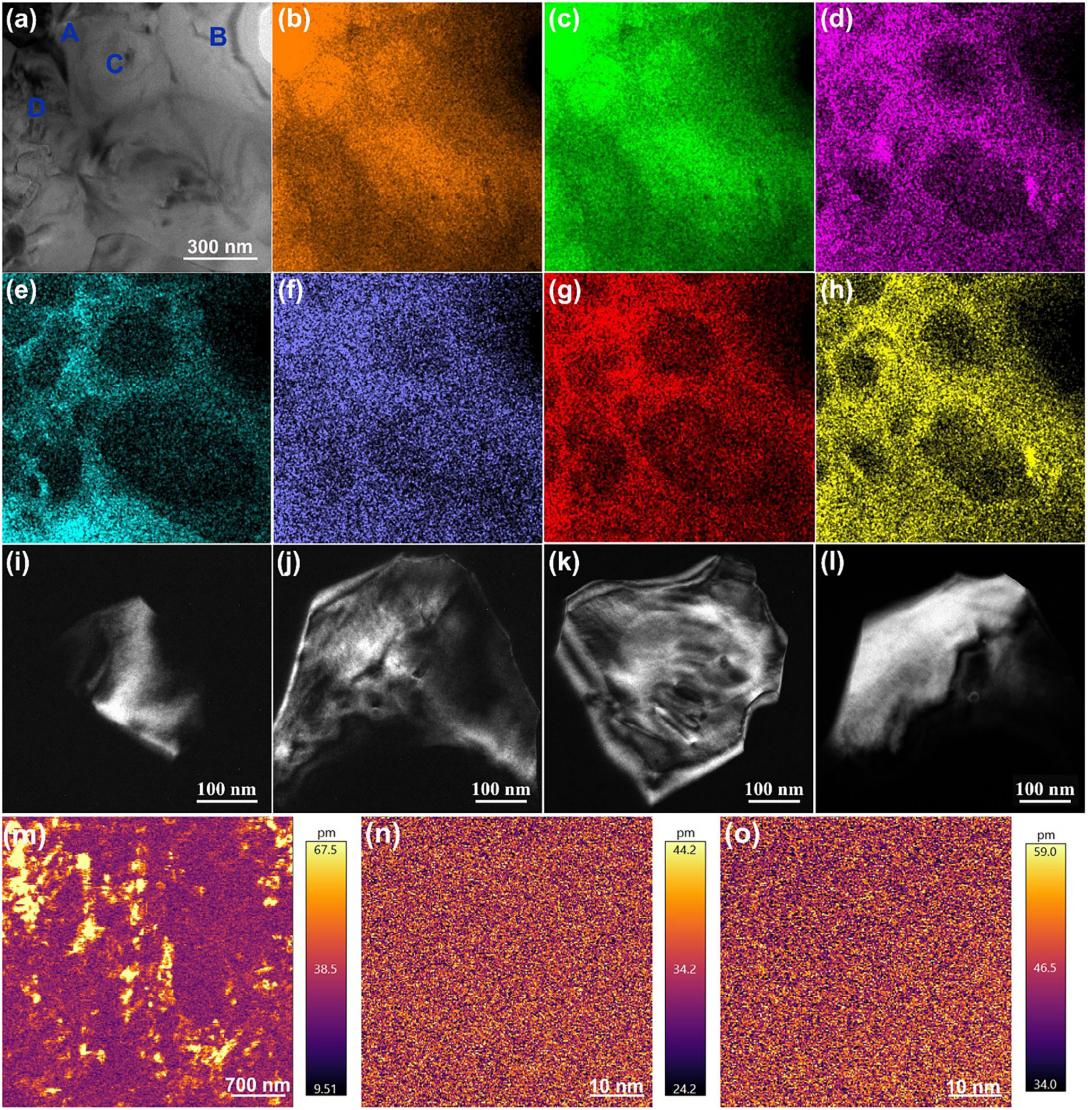
a) The bright‐field TEM image and corresponding EDS mappings of b) Ba, c) Ti, d) Ca, e) Zr, f) Bi, g) Na, h) Sn elements for the *x* = 0.16 SPS ceramic. i–l) Dark‐field TEM images of domain morphology for various grains in the *x* = 0.16 SPS ceramic. m–o) Out‐of‐plane PFM amplitude image of the *x* = 0.16 SPS ceramic.

To clarify the structural origin of the unique overall energy‐storage features in this work, the *x* = 0.16 SPS sample was characterized by using Transmission Electron Microscopy (TEM), as shown in **Figure** **4**. Through measurement of energy dispersive spectroscopy (EDS), mesoscopic chemical inhomogeneity can be easily observed throughout the whole SPS sample, as shown in Figure 4a‐h. The EDS result shows a significant inhomogeneous distribution of various elements at grain scale. By contrast, the CS sample shows an extremely‐uniform distribution of these elements, as displayed in Figure S3 (Supporting Information). The mesoscopic chemical inhomogeneity of the SPS ceramic should result from insufficient chemical element diffusion during the rapid sintering process of SPS,^[^ ^23^, ^29^ ^]^ as shown in Figure S4 (Supporting Information). Notably, no visual evidence of the local contrast related to any FE domains can be clearly found in the *x* = 0.16 SPS ceramic by means of conventional TEM owing to the extremely small size of nanodomains (Figure 4i–l). However, the PFM results suggest dissimilatory nanodomain morphologies in these chemically heterogeneous grains according to the detected significant differences in polarity, as shown in Figure 4m. By detecting smaller regions on two different grains with high and low piezoresponse, respectively, the observation of inconspicuous regions without any clear patterns further confirms the existence of high‐polarity and low‐polarity nanodomains in different grains. According to the phase diagram of the CS ceramics, the grains with high BT content and low BNS content, such as grain C and grain D, should possess weak dielectric relaxation characteristics and long‐range FE ordered domains. The formation of short‐range ordered nanodomains should be closely related to the remarkably reduced grain scale from CS ceramics to SPS ceramics, leading to the disrupted long‐range FE order owing to the enhanced local random fields.^[^ ^42^, ^43^ ^]^

**Figure 5:**
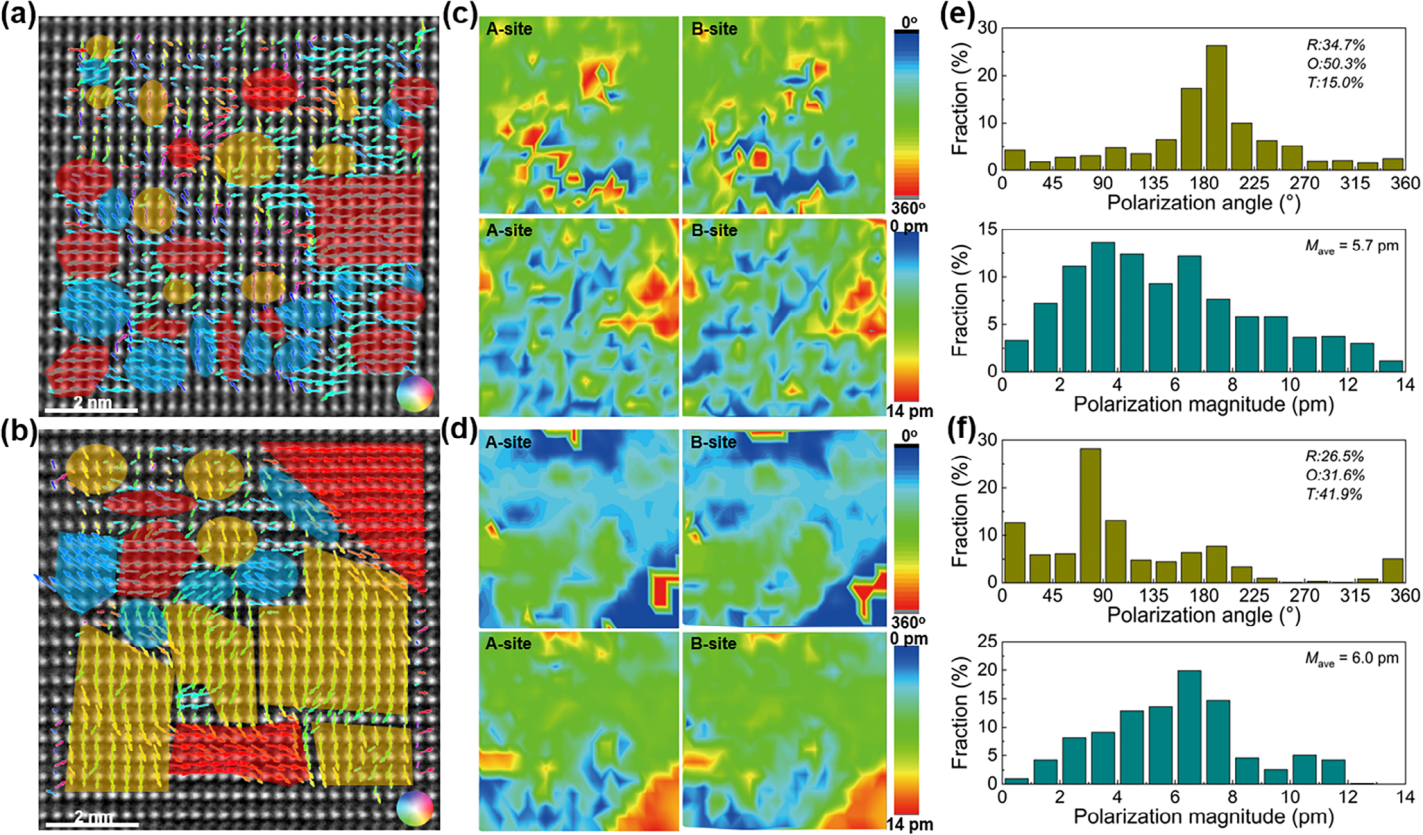
a,b) Atomic‐resolution HAADF STEM polarization vector images along [110]_c_ on two different grains. The red, orange, and blue areas represent the PNRs with O, T, and R symmetries, respectively. c,d) Polarization magnitude mapping and polarization angle mapping as well as e,f) the polarization magnitude and direction distribution of A/B‐site cations obtained from the HAADF images.

The diverse nanodomain architectures can be further investigated by analysis of high‐angle angular dark‐field (HAADF) performed on a scanning TEM (STEM), which is a more precise way to study the local symmetry of relaxors with nanodomains due to its atomic resolution. **Figures** **5**a,b demonstrate the HAADF images and corresponding atomic displacement vector along [110]_c_ of two chemically heterogeneous grains. The A‐site cations appear as the brighter spot, while B‐site cations show a weaker contrast. The results of the calculated off‐center displacements of A/B‐site cations are shown as color‐coded arrows according to their directions. The regions showing arrows with near‐zero polarization magnitude indicate the C phase, which can be revealed more clearly by the dark blue areas existing in Figure 4c‐d. When viewed along the <110> zone axis, the R, O, and T phases exhibit polarization vectors along the [110], [111], and [001] directions, respectively.^[^ ^1^, ^12^, ^13^, ^14^ ^]^ It can be found from Figure 5a‐b that the polarization angle distributes randomly in the studied regions, showing short range polarization ordering behavior and strong local polar fluctuations. Furthermore, both the two grains exhibit coexisting R‐O‐T‐C multiple local symmetries, despite the chemical inhomogeneity. However, the PNRs formed by regions with similar polarization orientations in the macroscopic pseudo‐C‐phase matrix exhibit a scale of 0.5–2 nm and 1–4 nm, respectively, in the two grains. The different domain size should be resulted from the chemical inhomogeneity, which also leads to the differentiated phase content and polarity, as evidenced by Figure 5e,f. The high‐BT‐content grains exhibit obviously reduced nonpolar regions and increased average polarization magnitude as compared with the low‐BT‐content grains.

**Figure 6:**
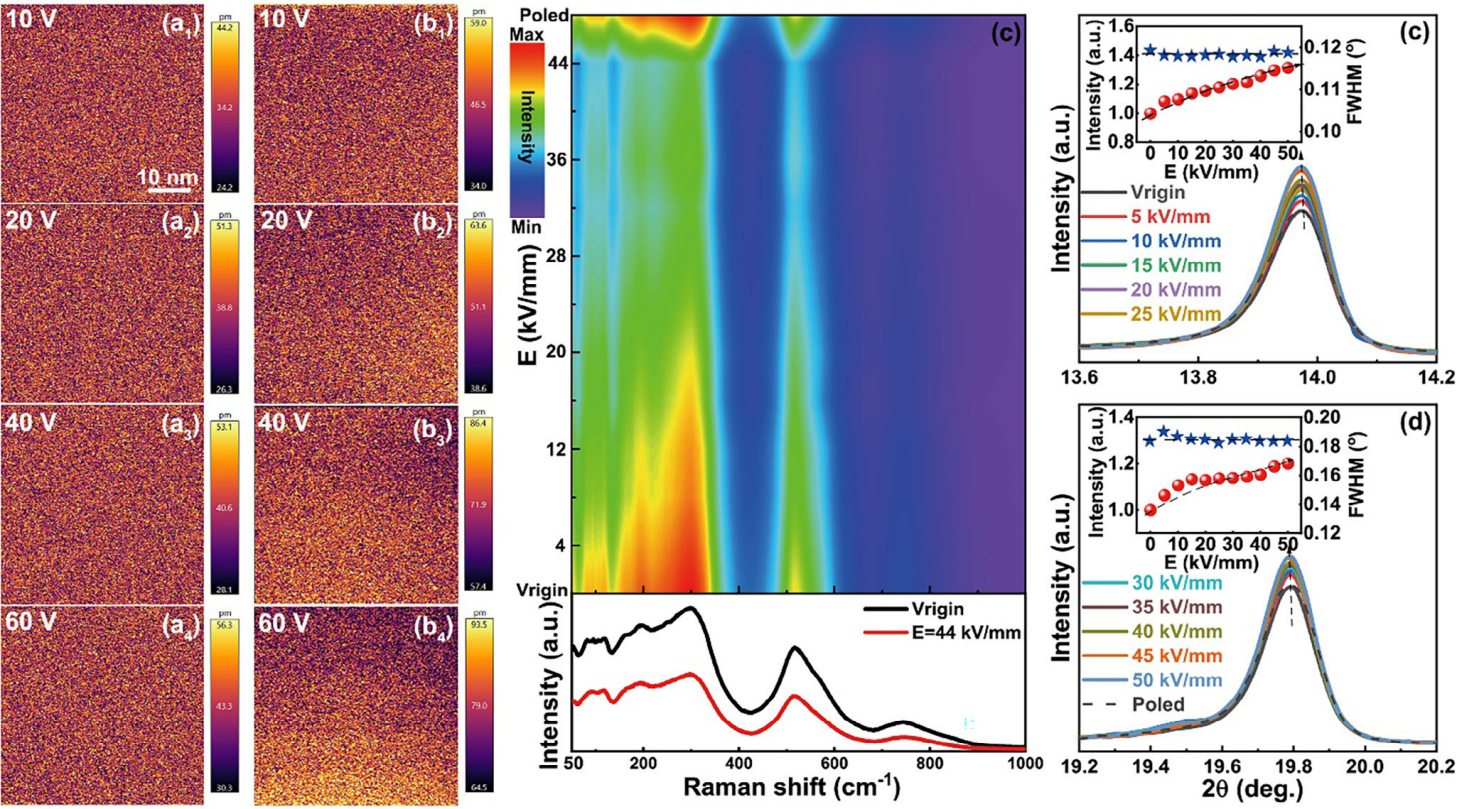
a,b) Out‐of‐plane PFM amplitude images of the *x* = 0.16 SPS ceramic measured at increasing external voltages. c) Raman spectra with changing external electric field for the *x* = 0.16 SPS sample and evolution of a,b) the (200)c and (220)c SXRD reflections.

To verify whether the polymorphic PNRs with different scales in diverse grains exhibit differentiated response behavior to external electric fields, the in situ multiscale structure evolution with electric fields is analyzed by PFM, Raman spectra, and SXRD. The PFM results measured at increasing external voltages are shown in **Figure** **6**d,e, which displays the domain evolution of two chemically heterogeneous grains. The enhancement of amplitude signals can be observed in both the two grains during increasing voltages, confirming the polarization reorientation with electric fields. However, the second grain with higher polarity shows a significantly faster increase in piezoresponse amplitude, and regions with obviously different contrast begin to appear at 60 V. By comparison, the first grain with lower polarity displays slowly increased piezoresponse amplitude, and no obvious regions can be found under various voltages. This indicates the heterogenous nanodomain switching behavior under applied electric fields of the two grains due to their different nanodomain scales, being responsible for linear‐like polarization‐field response. It is noteworthy that the formation of long‐range ordered FE microdomains under an electric field was not observed in both the two grains from in situ PFM results. This can be further confirmed by the Raman spectra and SXRD, as shown in Figure 6c‐d,e, respectively. For field‐dependent Raman spectra, the decreased intensity of Raman peaks with the increase of the electric field is related to the vertically applied electric field.^[^ ^20^, ^46^, ^47^ ^]^ No obvious change in both shape and number of Raman peaks can be found even under a high electric field up to 50 kV mm^−1^, suggesting that the long‐range FE order is not established. Moreover, for both (200)c and (220)c diffraction peaks in SXRD, the peak intensity exhibits a continuous increment while the full width at half maximum (FWHM) shows little change with electric fields, indicating a dominant contribution from the PNR reorientation in response to external fields since the PNR to microdomain transformation would result in an enhanced FWHM of the reflections.^[^ ^20^, ^44^, ^45^ ^]^ The above results suggest that the polymorphic PNRs with different scales in diverse grains would be switched and only undergo slight growth during increasing electric fields. This should be associated with the large random (electric and strain) fields, suppressing the transformation from relaxor to normal FEs. At last, both Raman spectra and SXRD basically remain the same before and after electric field loading, illustrating reversible variations under external electric fields.^[^ ^20^, ^44^, ^45^ ^]^

**Figure 7:**
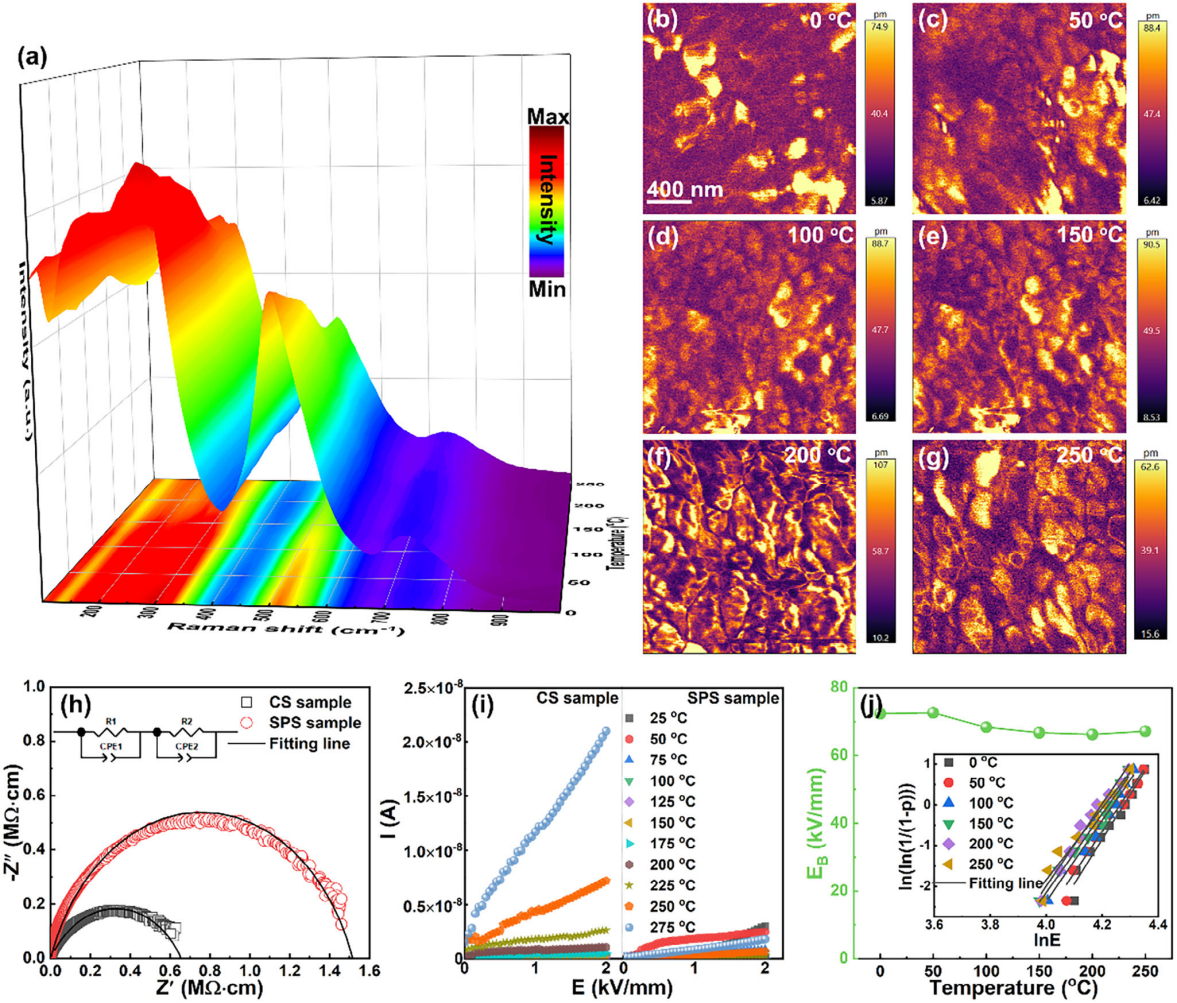
a) Evolution of the Raman spectra with changing temperature for the SPS sample. b–g) Out‐of‐plane PFM amplitude images of the SPS sample measured at 10 V on heating. h) Complex AC impedance and fitting semicircles at 500 °C,i) leakage current densities as a function of the external bias electric field for the CS and SPS ceramics. j) Weibull distribution and calculated E_B_ values of the SPS sample at different temperatures.^[^ ^50^ ^]^

The multiscale structure evolution with temperature is also studied to elucidate the excellent temperature stability of the energy‐storage properties. In situ Raman spectra measured on heating are shown in **Figure** **7**a, from which the slightly changed shape and intensity of the Raman peaks can be observed with increasing temperature from 0 °C to 150 °C. With further increasing temperature, the Raman peaks gradually widens and become smoother, indicating an enhanced local structure disorder and reduced unit cell polarity. However, the number of Raman peaks exhibits little change with temperature, suggesting an unchanged local symmetry within the wide temperature range. This should be associated with the complex stress conditions within the ultrafine grains, leading to the suppressed phase transformation with temperature.^[^ ^25^ ^]^ Furthermore, the temperature‐dependent PFM results measured at 10 V for the SPS samples are shown in Figure 7b–g. Two representative kinds of grains, one with high piezoresponse amplitude and another with low piezoresponse amplitude, can be clearly detected at 0 °C. All the grains show a significant enhancement of piezoresponse amplitude on heating before 250 °C, which should be associated with the enhanced nanodomain dynamic owing to the slightly reduced domain size and unit cell polarity. This is consisted with the results of Raman spectra, demonstrating an easier polarization reversal under external electric fields. As a result, the polarization hysteresis, which is already extremely low at near room temperature, is almost eliminated at high temperatures, as shown in Figure 2a. The decreased piezoresponse amplitude observed at 250 °C is suggested to result from the largely increased C phase content. Correspondingly, the *P_max_* shows a slightly decrease. Overall, the degradation of performance caused by the slightly decreased *P_max_* at high temperatures is compensated by the minimized polarization hysteresis. Thus, a near‐zero energy loss (≤ 2%) with a large *W_rec_* of ≥10 J cm^−3^ can be obtained at 50–250 °C. Furthermore, it is worth noting that the suppression of defect movement at high temperatures should be another essential reason for the high‐temperature stability of energy storage performance.^[^ ^13^, ^48^ ^]^ For numerous dielectric ceramics, the massive point defects especially for oxygen vacancies can become highly‐active free charge carriers at high temperatures, leading to the deterioration of *E_B_* and increment of polarization hysteresis.^[^ ^12^, ^13^, ^15^, ^17^ ^]^ Due to the rapid low‐temperature sintering process of SPS technology, the production of oxygen vacancies in the SPS ceramics can be greatly suppressed.^[^ ^28^, ^49^ ^]^ This can be confirmed by the XPS spectra of O1s for the *x* = 0.16 CS and SPS ceramics (Figure S5, Supporting Information), in which the content of the peak area related to the oxygen vacancy in the lattice decreases significantly from CS to SPS samples.^[^ ^49^ ^]^ Moreover, the significant reduction in grain size results in an increased content of grain boundary with high resistivity, as evidenced by the impedance spectroscopy in Figure 7h and Figure S6 (Supporting Information), which was analyzed by a a parallel R‖CPE equivalent circuit in the inset of Figure 7h.^[^ ^9^, ^39^ ^]^ The fitting results demonstrate that the conduction mechanism for the two ceramics is dominated by grain boundary. Moreover, the activation energy of the conductivity (*E_a_*), obtained from the temperature‐dependent resistivity through the Arrhenius equation *σ* = *σ_0_* exp(*E_a_*/*kT*), is displayed in Figure S6 (Supporting Information).^[^ ^39^, ^49^ ^]^ Both the increased *E_a_* and ground boundary resistivity would bring about impediment for the migration of oxygen vacancies.^[^ ^48^, ^49^ ^]^ The suppressed high‐temperature leakage current density and dielectric loss confirm the inhibition of defect motion at high temperatures, as shown in Figure 7i and Figure S7 (Supporting Information). This also contributes to a maintained high *E_B_* at high temperatures, providing a fundament for achieving excellent temperature‐insensitive energy storage properties.

## 3 Conclusion

In summary, an effective strategy of adjusting mesoscopically chemical homogeneity of polymorphic relaxor FEs is proposed to achieve breakthroughs in overall energy storage performances of dielectric ceramic capacitors, which has been successfully realized in BCZT‐BNS lead‐free ceramics. Diversified nanodomains featuring similar multiple local symmetries but different scales were constructed in chemically heterogeneous submicron grains, resulting in a relatively continuous polarization switching with increasing the applied electric field from diverse grains. This endows a linear‐like *P‐E* loop with a high *P_max_* and a near‐zero hysteresis, leading to a giant *W_rec_* ≈ 15.4 J cm^−3^ with an ultrahigh *η* ≈ 93.2% under 78 kV mm^−1^ at room temperature. Furthermore, owing to the high thermal stability of the nanodomain structure characteristics and inhibition of high‐temperature defect motion in ceramics, the state‐of‐the‐art high‐temperature energy‐storage performances of *W_rec_* = 10.4 ± 5% J cm^−3^ and *η* = 96 ± 3% can be obtained at 65 kV mm^−1^ within an ultra‐broad temperature range of 0–250 °C, demonstrating great petential for applications in high‐temperature pulsed power systems. The proposed approach in this work should be applicable to the development of novel high‐performance dielectric materials.

## 4 Experimental Section

### Material Synthesis

The (1‐*x*)BCZT‐*x*BNS perovskite powders were synthesized by a conventional solid‐state reaction method using high‐purity raw materials (> 99%) of BaCO_3_, CaCO_3_, Na_2_CO_3_, Bi_2_O_3_, TiO_2_, ZrO_2,_ and SnO_2_. The weighed raw powders were mixed at first, and then ball milled in ethanol with zirconia balls for 6 h. Next, the dried powders were calcined at 1000 °C for 6 h in a covered alumina crucible. In the case of the CS samples, pellets 10  mm in diameter were pressed and then sintered in sealed crucibles at 1330–1360 °C for 2 h. In the case of the SPS samples, the powders were placed in a graphite die and sintered at 1000 °C for 8 min with an SPS apparatus (Labox‐675, Sinter Land, Japan) at the heating rate of 100 °C min−1. Obtained powders were pressed into pellets with a diameter ≈10 mm and sintered in sealed crucibles at 1240–1280 °C for 2 h. During SPS, a mechanical uniaxial pressure of 50 MPa was applied to the sample. After sintering, the SPS samples were thermally treated at 800 °C for 6 h in air to remove the carbon contamination. Finally, the sintered ceramic samples were grinded and polished to ≈80 µm in thickness and sputtered with gold electrodes with a diameter of ≈1.0 mm for the measurements of *P‐E* loop, *E_B_*, and charge–discharge properties.

### Dielectric Measurements

Temperature/frequency‐dependent dielectric properties and impedance test were used by an LCR meter (E4980A, Agilent Technologies, Inc., USA) connected with a high/low‐temperature probing stage (HCT1821, Tongguo technology, China) and an impedance analyzer (E4990A, Agilent Technologies, Inc., USA) connected with a high‐temperature probing stage (GWDS003, Tongguo Technology, China), respectively.

### Ferroelectric Measurements

An FE measurement system (TF Analyzer 2000E, aixACCT Systems GmbH, Germany) connected with a high‐temperature probing stage (HFS600E‐PB2, Tongguo technology, China) was used to measure the *P‐E* loops.

### Energy‐Storage Properties

The energy release properties were investigated through a charge‐discharge platform (CFD‐003, Tongguo Technology, China).

### Leakage Current

The leakage current of the ceramics was measured by an electrometer/high resistance meter (6517B, Keithley, USA) connected with a pyroelectric testing system (HCT1801, Tongguo technology).

### Dielectric Breakdown Test

The *E_B_* measurements were carried out on a voltage‐withstand test device (BDJC‐50KV, Beiguangjingyi Instrument Equipment Co. Ltd., China).

### Scanning Electron Microscopy

A field‐emission scanning electron microscope (FE‐SEM, SU8020, JEOL, Japan) was used to observe the grain morphology on the polished and thermally etched surface of the ceramics.

### Synchrotron X‐Ray Diffraction

To analyze the average phase structures, the full profiles of SXRD were carried out at beamline 14B1 (λ = 1.2398 Å, 10 keV) at the Shanghai Synchrotron Radiation Facility (SSRF). Rietveld refinement was performed on full SXRD data using GSAS software.

### Raman Spectra

A Raman spectrometer (LabRam HR Evolution, HORIBA JOBIN YVON, Longjumeau Cedex, France) connected with a high‐temperature probing stage (HFS600E‐PB2, Linkam Scientific Instruments, UK) was used to collect Raman spectra on well‐polished samples.

### Piezoresponse Force Microscopy

The PFM (AsylumResearch MFP‐3D Origin+, Oxford Instruments, UK) connected with a high voltage amplifier was used to analyze the domain morphology evolution with temperature and bias voltage.

### Transmission Electron Microscopy

The atomic‐scale STEM HAADF images were performed on a probe‐corrected Hitachi HF5000 at 200 kV, while the domain morphology observations and EDS measurements were carried out on a JEOL JEM‐F200 microscope at 200 kV. Chemical composition mapping with EDS was performed in JEOL JEM‐F200 at 200 kV along with two integrated silicon‐drift EDS detectors. Specimens for STEM measurements were prepared based on a sintered ceramic through a conventional approach combining mechanical thinning and finally Ar^+^ ion‐milling in a Gatan PIPS II (Gatan Inc., Pleasanton, CA).

### Statistical Analysis

Grain size distribution analysis was systematically conducted using Nano Measurer software. A total of 200 grains were statistically evaluated to ensure representative sampling, yielding an average grain size with a relative deviation below 5%. For Weibull distribution characterization, the counted number of the samples for Weibull distribution analysis was 10, and the deviation of the mean *E_B_* values was <1%.

## Conflict of Interest

The authors declare no conflict of interest.

## Supporting information



Supporting Information

## Data Availability

The data that support the findings of this study are available from the corresponding author upon reasonable request.
