# Supplementary material for: Superior Capacitive Energy Storage of BaTiO3‐Based Polymorphic Relaxor Ferroelectrics Engineered by Mesoscopically Chemical Homogeneity
Source: Adv Sci (Weinh). 2025 May 24;12(27):2502916. doi: 10.1002/advs.202502916 (PMC12279175; doi:10.1002/advs.202502916)

Supporting Information

Superior Capacitive Energy Storage of BaTiO_3_-Based Polymorphic Relaxor Ferroelectrics Engineered by Mesoscopically Chemical Homogeneity

*Aiwen Xie, Ziyi Yu, Junwei Lei, Yi Zhang, Ao Tian, Xuewen Jiang, Xinchun Xie, Yuewei Yin, Zhenqian Fu*, Xiaoguang Li*, Ruzhong Zuo**

**Table S1.** Refined structural parameters of *x* = 0.16 CS and SPS ceramic powders.

| *Ceramic* | Space group | Fraction  (%) | Lattice parameters | V  (Å^3^) | *R_wp_*  (%) | *χ^2^* |
| --- | --- | --- | --- | --- | --- | --- |
| CS | *R3m*  *Amm2*  *P4mm*  *Pm3m* | 30.2  11.4  22.8  35.6 | a=b=5.6721 Å, c=6.9248 Å, α=β=90^o^, γ=120^o^  a=4.0120 Å, b=5.6742 Å, c=5.6629 Å, α=β=γ=90^o^  a=b=4.0186 Å, c=4.0251 Å, α=β=γ=90^o^  a=b=c=4.0230 Å, α=β=γ=90^o^ | 192.94  128.917  65.003  65.109 | 6.6 | 1.28 |
|  |  |  |  |  |  |  |
| SPS | *R3m*  *Amm2*  *P4mm*  *Pm3m* | 28.8  10.6  23.6  37.0 | a=b=5.6800 Å, c=6.9184 Å, α=β=90^o^, γ=120^o^  a=4.0202 Å, b=5.6708 Å, c=5.6558 Å, α=β=γ=90^o^  a=b=4.0198 Å, c=4.0389 Å, α=β=γ=90^o^  a=b=c=4.0340 Å, α=β=γ=90^o^ | 193.3  128.939  65.247  65.646 | 6.8 | 1.33 |

**Figure S1.** a-b) Temperature-dependent dielectric properties of (1-*x*)BCZT-*x*BNZ CS ceramics. c) Rietveld SXRD refinement results for the *x* = 0.16 CS ceramic powders. d) *ΔT_relax_* and *γ* values as a function of BNS content. e) Room-temperature *P-E* loops of (1-*x*)BCZT-*x*BNZ CS ceramics measured at 7 kV mm^-1^ and 10 Hz. f) Temperature/frequency-dependent *ε_r_* of the *x* = 0.16 SPS ceramics.


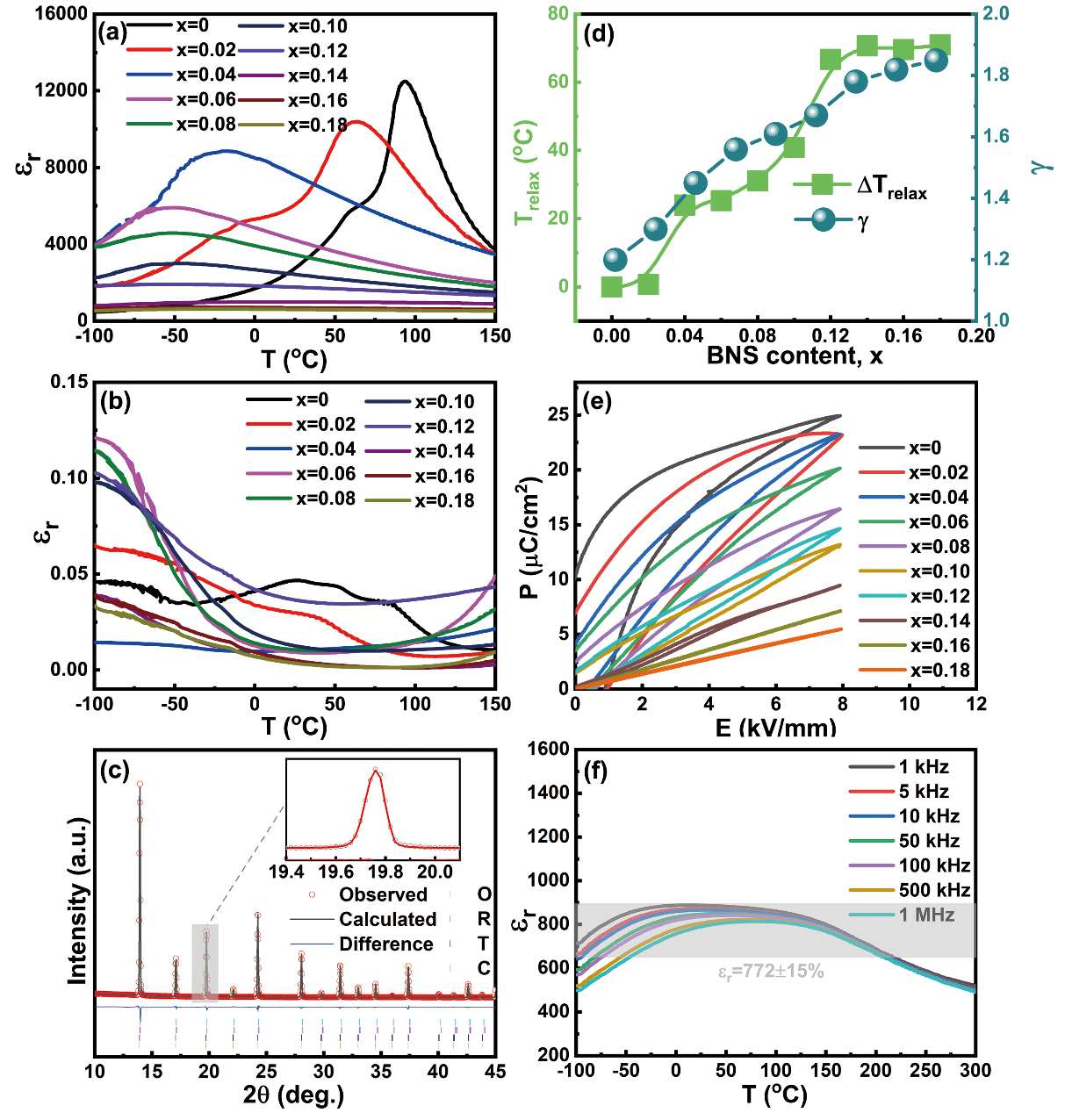


**Figure S2.** a) Room-temperature pulsed overdamped discharging current curves and b) corresponding *W_D_-t* curves of the *x* = 0.16 SPS sample at a fixed load resistance of 300 Ω under various electric fields. c) Room-temperature pulsed underdamped discharging current curves of the *x* = 0.16 SPS sample under various electric fields. d) The *P_D_*, *W_D_* and *t_0.9_* values as a function of the applied electric field for the *x* = 0.16 SPS sample. e) A comparison of *W_D_* and *P_D_* values between the *x* = 0.16 SPS sample and various recently reported lead-free bulk ceramics (NN: NaNbO_3_; AN: AgNbO_3_; BNT: (Bi_0.5_Na_0.5_)TiO_3_; BKT: (Bi_0.5_K_0.5_)TiO_3_; BF: BiFeO_3_; ST: SrTiO_3_).[4-13,16,18-22,30-35]

.


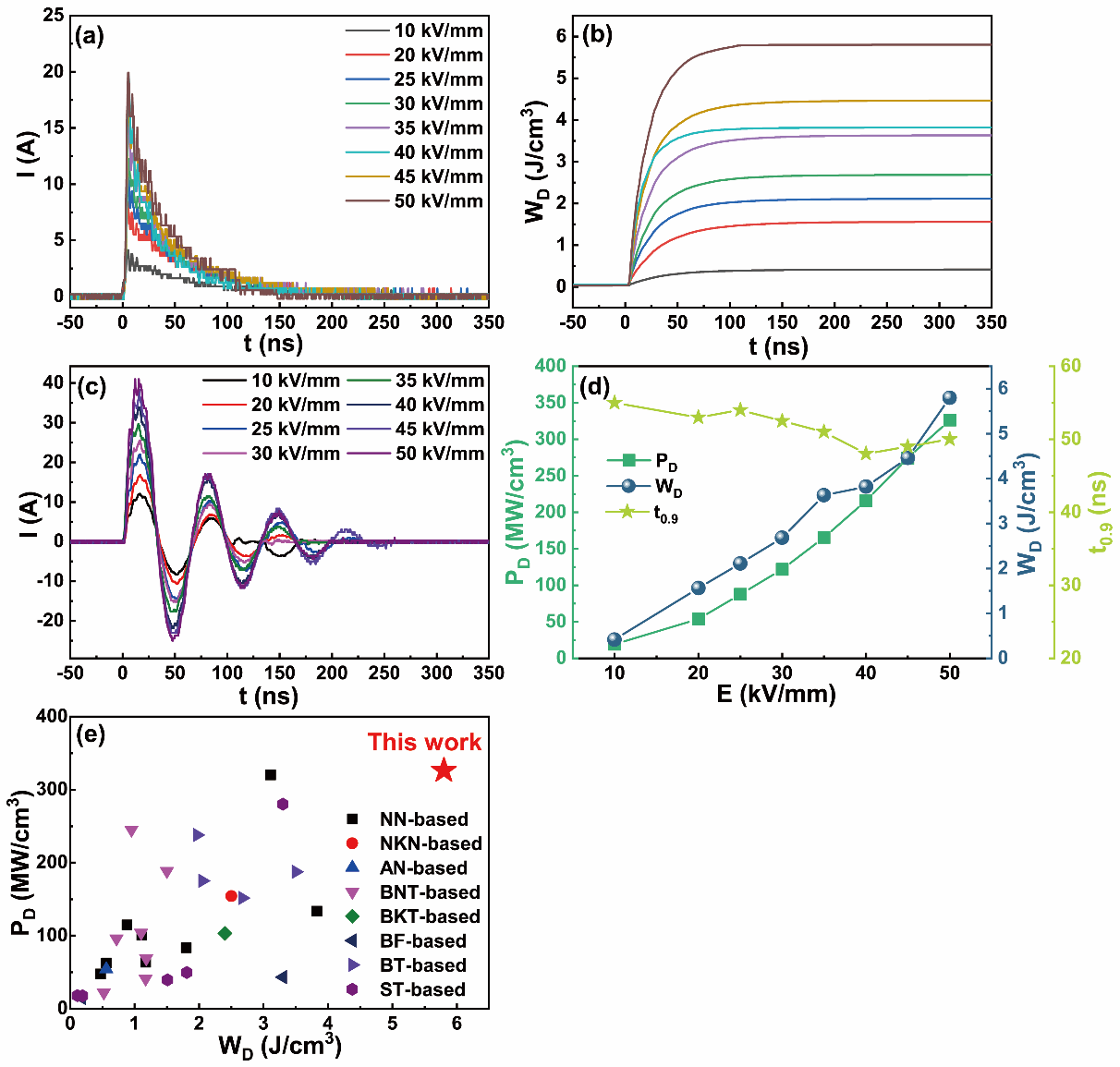


**Figure S3.** The a) domain micrographs, b) IFFT and c) EDS mappings of *x* = 0.16 CS ceramics.


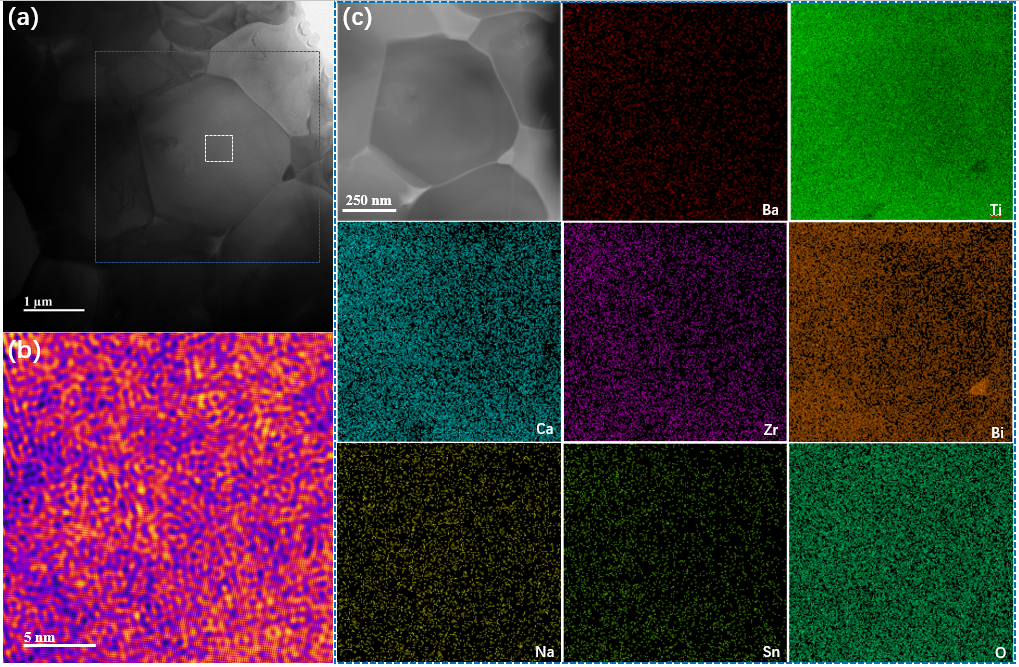


**Figure S4.** EDS mappings of the *x* = 0.16 calcined powders.


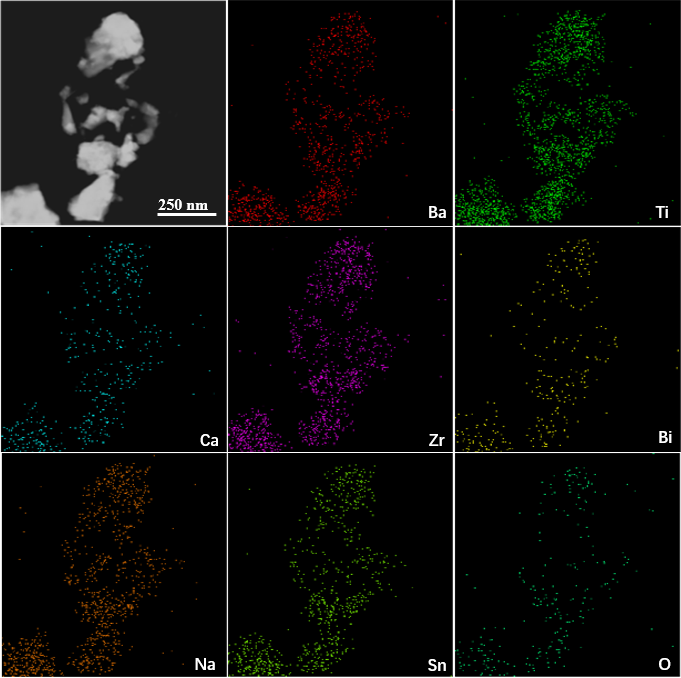


**Figure S5.** XPS spectra showing the O1s spectral region for the *x* = 0.16 a) CS and b) SPS ceramics.

**
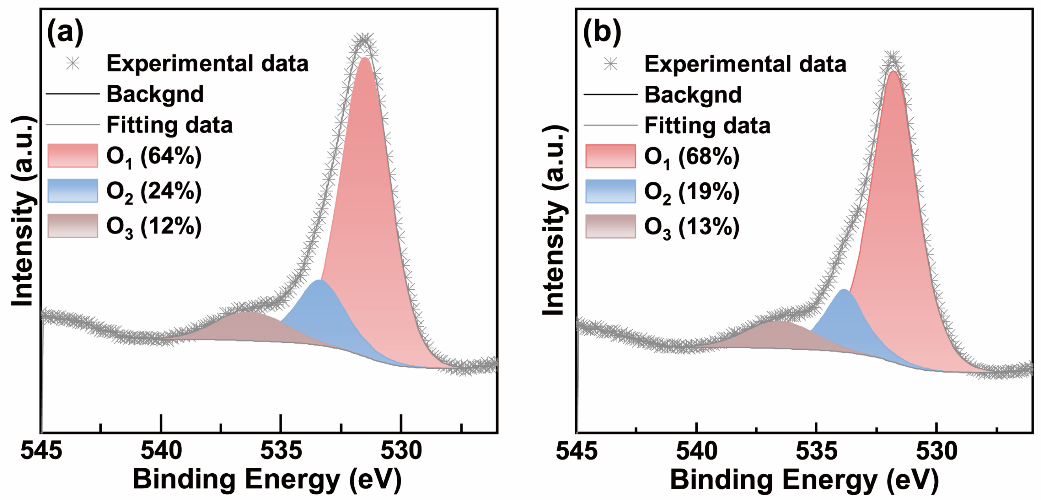
**

**Figure S6.** Complex AC impedance measured at different temperatures for the *x* = 0.16 a) CS and b) SPS ceramics.


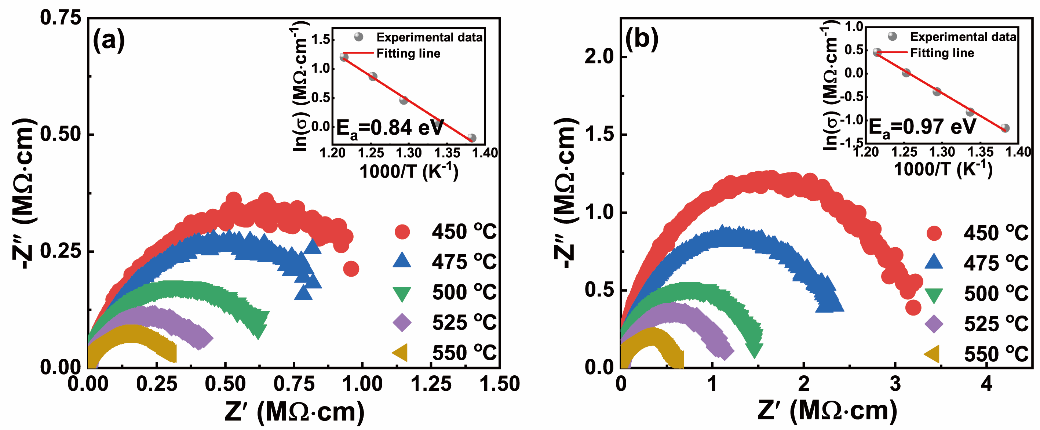


**Figure S7.** Temperature-dependent tanδ of the *x* = 0.16 SPS ceramics.


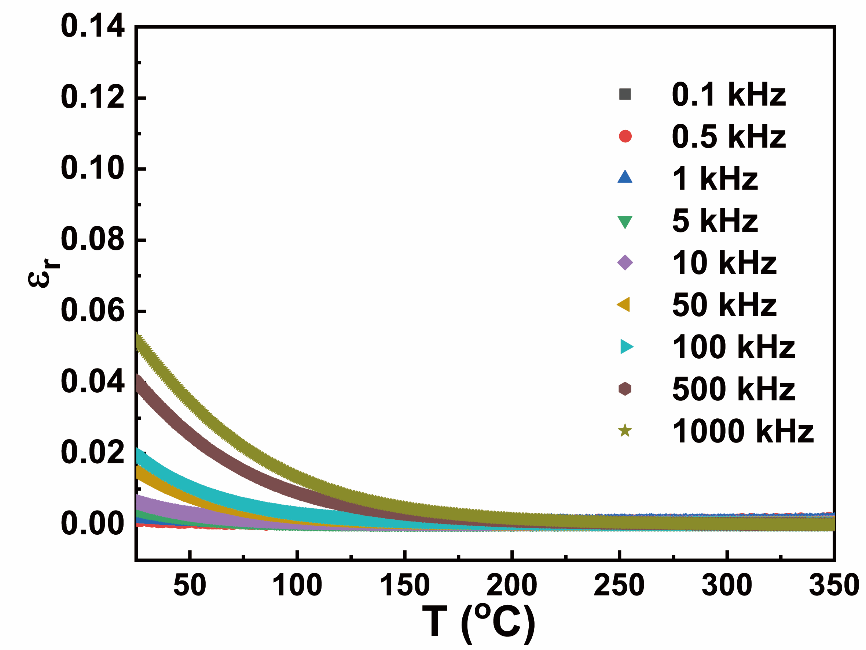

Supplement: Supplementary file 1 — Supporting Information [file ADVS-12-2502916-s001.docx]
